# Derivation and validation of a risk-factor model for detection of oral potentially malignant disorders in populations with high prevalence

**DOI:** 10.1038/sj.bjc.6605778

**Published:** 2010-07-13

**Authors:** H K Amarasinghe, N W Johnson, R Lalloo, M Kumaraarachchi, S Warnakulasuriya

**Affiliations:** 1 Dental Institute, Maharagama, Colombo 10280, Sri Lanka; 2 Griffith Health Institute, Gold Coast Campus, Griffith University, Queensland 4222, Australia; 3 School of Dentistry and Oral Health, Gold Coast Campus, Griffith University, Queensland 4222, Australia; 4 Department of Oral Medicine and Experimental Oral Pathology, King's College Dental Institute, London SES 9RS, UK

**Keywords:** oral potentially malignant disorders, oral cancer, risk factors, screening, socioeconomic status, Sri Lanka

## Abstract

**Background:**

Oral and pharyngeal cancers constitute the sixth most common type of cancer globally, with high morbidity and mortality. In many countries, most cases of oral cancer arise from long-standing, pre-existing lesions, yet advanced malignancies prevail. A new approach to early detection is needed. We aimed to validate a model for screening so that only high-risk individuals receive the clinical examination.

**Methods:**

A community-based case–control study (*n*=1029) in rural Sri Lanka assessed risk factors and markers for oral potentially malignant disorders (OPMD) by administering a questionnaire followed by an oral examination. We then developed a model based on age, socioeconomic status and habits of betel-quid chewing, alcohol drinking and tobacco smoking, with weightings based on odds ratios from the multiple logistic regression. A total, single score was calculated per individual. Standard receiver-operator characteristic curves were plotted for the total score and presence of OPMD. The model was validated on a new sample of 410 subjects in a different community.

**Results:**

A score of 12.0 produced optimal sensitivity (95.5%), specificity (75.9%), false-positive rate (24.0%), false-negative rate (4.5%), positive predictive value (35.9%) and negative predictive value (99.2%).

**Conclusion:**

This model is suitable for detection of OPMD and oral cancer in high-risk communities, for example, in Asia, the Pacific and the global diaspora therefrom. A combined risk-factor score of 12.0 was optimal for participation in oral cancer/OPMD screening in Sri Lanka. The model, or local adaptations, should have wide applicability.

Oral and pharyngeal cancer is the sixth most common cancer in the world, with an annual global estimated incidence of about 275 000 for oral and 130 300 for pharyngeal cancers in the year 2002, excluding salivary neoplasms, malignant neoplasms of the nasopharynx and of the pyriform sinus – two-thirds of these occur in developing countries (Parkin *et al*, 2005). A particularly high incidence is observed in the Indian sub-continent, which accounts for one-third of the world's burden of oral cancer (Parkin *et al*, 2001). Rates are also high in Melanesia and other Pacific islands, in parts of Southeast (SE) Asia (Parkin *et al*, 2001), in Taiwan and some provinces of mainland China where areca nut and/or betel-quid chewing habits are common (Jeng *et al*, 2001; Gupta and Warnakulasuriya, 2002; Zhang and Reichart, 2007). The incidence of cancer of the oral cavity and oropharynx (excluding salivary neoplasms) in Sri Lanka, standardised to the world population, in the year 2005, was 14.1 and 3.8 per 100 000 in males and females, respectively (National Cancer Control Programme Sri Lanka, 2009).

Oral cancer is often preceded by so-called ‘premalignant lesions’ or ‘potentially malignant lesions and conditions’. A recent workshop conducted by the WHO Collaborating Centre for Oral Cancer and Precancer in London has recommended the term oral potentially malignant disorders (OPMD) (Warnakulasuriya *et al*, 2007). The global prevalence of OPMD is reported to be between 1 and 5% (Napier and Speight, 2008). A high prevalence of OPMD is reported from South and East Asia with male preponderance (Chung *et al*, 2005; Ariyawardana *et al*, 2007), and with malignant transformation rates of over 2% per year (Napier and Speight, 2008). According to the Sri Lankan National Oral Health Survey 2002/2003, it is estimated that more than 284 000 people are alive with OPMD, a prevalence of 3.4% (Ministry of Health Sri Lanka, 2009).

The nature and prevalence of risk factors for OPMDs differ by country and region. In the developing world, tobacco and areca nut used either singly or in various combinations of ‘betel quid’ or ‘pan’ (Chung *et al*, 2005; Ariyawardana *et al*, 2007), account for the vast majority of the most common OPMD, leukoplakia. We have recently shown that, in Sri Lanka, the population attributable risks for OPMD of daily betel-quid chewing and of regular consumption of alcohol can be estimated at 84 and 25%, respectively (Amarasinghe *et al*, 2010). A diet poor in antioxidant vitamins and trace elements also constitutes a significant risk factor for oral cancer (Maher *et al*, 1997; Nagao *et al*, 2000; Warnakulasuriya, 2009a). Further, recent meta-analyses of the available literature have shown that socioeconomic status (SES) can be considered a significant risk marker for oral cancer, presumably as a surrogate for poor diet, and for heavier use of areca, tobacco and perhaps alcohol (Conway *et al*, 2008; Warnakulasuriya, 2009b).

In South and Southeast Asia, screening for OPMD has been carried out at various levels and in different settings, ranging from whole communities, targeted to high-risk groups and opportunistically in clinical environments (Warnakulasuriya *et al*, 1984; Sankaranarayanan *et al*, 2005; Amarasinghe, 2007). Of these, screening a whole community can be achieved effectively and economically by using an existing health workforce, especially those based within the community itself. A study conducted by one of us in Sri Lanka in the early 1980's using Primary Health Care (PHC) staff in detection of OPMD and oral cancer reported a sensitivity of 89% (Warnakulasuriya *et al*, 1984). As a result, this approach has been included in the National Health Policy of Sri Lanka since 1990 (Ministry of Health Sri Lanka, 1990). In spite of this, no sustainable screening programmes have been implemented and Sri Lankan hospitals are reporting an increased proportion of patients with oral cancer, presenting with advanced, often incurable, disease. This is most unfortunate in light of the evidence from the extensive Trivandrum Oral Cancer Screening study, which shows that deaths can be prevented in high-risk communities by such programmes (Sankaranarayanan *et al*, 2005).

We have determined that the main obstacles to effective oral cancer screening over the intervening three decades include: the lack of adequate guidelines for PHC staff, particularly concerning which individuals should be examined; an excessive workload, including their duties with mothers and babies, and with immunisation programmes; devolution of all vertical preventive programmes to the provincial level; and lack of quality continuing education and assessment systems for health workers (Amarasinghe, 2007). As a possible solution to these problems, we have developed a model designed to identify, in advance, individuals at high risk for oral cancer and for OPMD who can then be targeted for oral examination and for focused preventive measures. Our approach is consonant with the Crete Declaration on Oral Cancer Prevention (Peterson, 2005).

## Materials and methods

This study was carried out in two phases with ethical approval from the ethics review committee, Faculty of Medicine, University of Colombo. Phase 1 consisted of a community-based case–control study in the Sabaragamuwa province of rural Sri Lanka. This is located between the western and central provinces and has two districts, Ratnapura and Kegalle. The population characteristics, ethnic mix and socioeconomic diversity is described in detail elsewhere (Amarasinghe, 2008). One administrative area defined by the Medical Officer of Health (MOH) for the district was randomly selected from each district. Ratnapura district has 15 MOH areas, whereas Kegalle district has 10 (Ministry of Health Sri Lanka, 2003). The selected MOH areas contained 42 public health midwife (PHM) areas covering villages and tea and rubber estates – these are considered as cluster units. Of these, 14 clusters were selected using the probabilities proportionate to size sampling technique, with deliberate oversampling of the estate sector. Using a house-to-house method, a total of 1029 willing subjects over 30 years of age were recruited over a 1-year period starting from November 2006.

With the informed signed consent of subjects, trained PHMs administered questionnaires designed to gather sociodemographic and lifestyle information, including details of betel-quid chewing, smoking and consumption of alcohol. Habits were defined in terms of never, ever, past, occasional and daily, as that used in several states in the eastern USA (Morse *et al*, 2007). Information on occupation and education was amalgamated to represent the SES of the subjects. Anthropometric measurements of height and weight were obtained to calculate body mass index (BMI). Details of diet were obtained, from which the protective effects of fruit and vegetables are reported elsewhere (Amarasinghe, 2008).

A visual oral soft tissue examination was carried out on each subject by the senior author (HKA) for identification of OPMD and any other abnormalities using mouth mirrors both to reflect light and the soft tissues and to examine inaccessible areas of the mouth. The examiner was blinded to the risk-factor status. The diagnostic criteria for the detection of OPMD, namely, leukoplakia, erythroplakia, oral submucous fibrosis (OSF) and lichen planus, were based on the recommendations of the WHO (Axell and Rundquist, 1987; Warnakulasuriya *et al*, 2007). Other oral mucosal abnormalities were defined according to the workshop held in Kuala Lumpur, Malaysia in 1996 (Zain *et al*, 1999). HKA has extensive experience of such examinations, and calibrations carried out in 2006 showed high-*κ* agreements with a number of studies carried out by other experienced oral surgeons and oral medicine practitioners (Amarasinghe, 2008).

Subjects with OPMD were taken as cases and those free of both OPMD and any other oral mucosal disease as controls. (Chewers mucosa, quid-induced lichenoid reactions, smoker's keratosis, denture stomatitis, angular cheilitis and oral manifestations of anaemia were considered as other oral mucosal diseases.)

### Statistical methods for Phase 1

Questionnaire and clinical data were recorded on paper, then entered into and analysed by the Statistical Package for Social Sciences (SPSS) Version 17 software package (SPSS inc., IBM, Chicago, IL, USA). Correspondence analysis was used to combine information on occupation and education to represent the SES of the subjects. This was the average of the two scores per individual. The range of scores obtained was then divided into terciles and each individual was placed within one of these bands (Zurriaga *et al*, 2004). Relationships between two categorical variables were tested by *χ*^2^-test. A multivariate logistic regression analysis, including in the model only variables that were statistically significant in the univariate analysis, was used to obtain effect estimates of the potential risk factors on OPMD (Table 1).

Factors to be included into the risk-factor model were based on the results of Phase 1. The risk indicators of age and SES, and the risk factors of betel-quid chewing, tobacco smoking and heavy alcohol drinking were selected. Gradients for each factor were given a score derived from the adjusted odds ratios (ORs) obtained, and were rounded to the nearest whole number to simplify the task for field workers.

A receiver-operator characteristics (ROC) curve was plotted against sensitivity and false-positive rate (FPR) to produce a cutoff point for presence of OPMD, excluding lichen planus. The main reason for excluding subjects with lichen planus in the OPMD group is because the aetiology of this condition, although still poorly understood, is not related to the risk factors included in our risk-factor model (Sugerman *et al*, 2002; van der Meij *et al*, 2003; Lodi *et al*, 2005; Ismail *et al*, 2007). Because individuals with oral lichen planus and other mucosal diseases are present in the test community, and will be present in other communities to which our risk-factor model might be applied, we have also analysed the data with these disorders included.

The area under the curve (AUC) represents the probability that a random test result will be ranked correctly, as to disease state. Theoretically, a diagonal line would be a score predicting not better than a random guess. Thus, if the AUC is 0.5 or less, the test has no value. The closer the area approaches 1.0, the greater the significance and utility of the test.

In Phase 2, the validation study was carried out in selected PHM areas of the Maharagama MOH, within the suburban population of the Colombo district, and in a rural population in selected PHM areas of the Bulathkohupitiya MOH area in the Kegalle district of Sri Lanka, over a 4-month period from November 2008. These two MOH areas were selected because of their ready accessibility, and the existence of on-going oral cancer control activities. The responsible PHM and a team of up to 10 volunteers advertised, during house-to-house visits, the opportunity for an oral/dental examination and especially encouraged betel and/or tobacco users to attend. Interviews and examinations were conducted locally, the latter using portable dental chairs and headlights. A total of 410 subjects over 15 years of age were recruited.

### Statistical methods for Phase 2

Data were entered and analysed by SPSS as in Phase 1. Each subject was given a score according to the risk-factor model described earlier, and this was compared with the results of the screening examination. The ROC curves were again plotted.

## Results

### Phase 1

A total of 101 cases of previously undiagnosed OPMD were detected by screening. In addition, there were four cases of oral cancer, one newly diagnosed and three under treatment and one case of treated leukoplakia; these were excluded from the analysis. A total of 195 subjects had another oral mucosal disease as defined earlier. There were 728 subjects without any mucosal abnormalities and these were designated as controls for the case–control analysis. When weighted for age and place of residence – namely, village or estate – this represents a prevalence of 11.3% for OPMD (Amarasinghe *et al*, 2010).

The risk of occurrence of an OPMD and its statistical significance, according to the logistic regression analysis, is shown in Table 1. Crude ORs were significant for the risk indicators of sex, age and SES, and for the risk factors of betel-quid chewing, smoking, BMI, consumption of alcohol and for intake of *β*-carotene containing fruits and vegetables. Betel-quid chewing and consumption of alcohol are the only statistically significant characteristics remaining after controlling for the other factors. After controlling for all other variables, the adjusted OR for daily chewers was 10.1 (95% CI: 3.4–29.7), with a strong dose–response relation. When considering the consumption of alcohol and risk of OPMD, the adjusted OR for weekly drinkers was 2.7 (95% CI: 1.2–6.3).

### Derivation of the risk factor model

Among the lifestyle risk factors, the most critical variables that differentiate disease from non-disease are betel-quid chewing and alcohol use (Table 1). The scores for these were, as described above, derived from the adjusted ORs obtained in Phase 1. Thus, for betel quid, non-chewers scored zero for this parameter, those chewing less than three quids per day scored 2.5 and those chewing three or more quids per day scored 16 (Table 2). Similarly for alcohol use, non-drinkers scored zero, past/occasional drinkers 1 and daily/weekly drinkers 3. Non-smokers were given a score of zero and ever-smokers a score of one. The SES scores were dichotomised to zero for high and 3 for middle and low. For the latter two risk factors/indicators, these values were also taken from the adjusted OR's even though these were not statistically significant, because they have clear importance in many published studies. The weighting for age was established from the literature (Fisher *et al*, 2005) and from our unpublished data because Phase 1 did not contain subjects below 30 years of age. The aim was to create a single score that can be easily calculated by adding weighted scores for each factor for each subject. Such scoring systems are currently gaining increasing utility in other health fields, such as cardiology and anaesthesiology (Eberhart *et al*, 2004; Pepe *et al*, 2004; Sullivan *et al*, 2004).

### Risk score analysis

**Figure 1:**
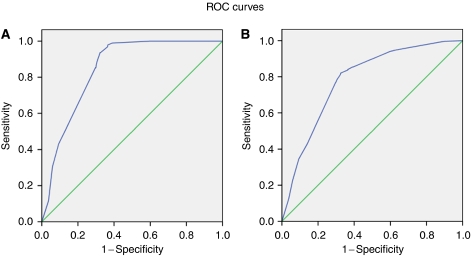
(**A**) The ROC curve for risk-factor model score and the presence of OPMD (except lichen planus) in Phase 1 study. (**B**) The ROC curve for risk-factor model score and the presence of all oral mucosal disorders in Phase 1 study.

Using all the risk factors and indicators in the model, a ROC curve was plotted for total score assigned to each subject and the presence of OPMD, excluding lichen planus (Figure 1A). A cutoff value of 12 yields maximum length to the diagonal line with an AUC of 0.84 (95% CI: 0.81–0.87), a sensitivity of 93.7%, a specificity of 67.7%, a FPR of 32.3%, a false-negative rate (FNR) of 6.3%, a positive predictive value (PPV) of 27.5% and a negative predictive value (NPV) of 98.8% (Table 3).

When all oral mucosal diseases are plotted against the full range of risk indicators and risk factors (Figure 1B), the same optimal cutoff point of 12 emerges, with an AUC of 0.78 (95% CI: 0.75–0.81), a sensitivity of 81.1%, a specificity of 67.7%, a FPR of 32.3%, a FNR of 18.9%, a PPV of 50.9% and a NPV of 89.6% (Table 4).

### Phase 2

**Figure 2:**
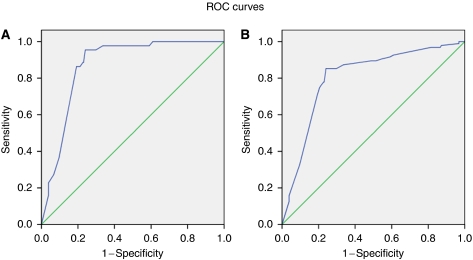
(**A**) The ROC curve for risk-factor model score and the presence of OPMD (except lichen planus) in Phase 2 validation study. (**B**) The ROC curve for risk-factor model score and the presence of all oral mucosal disorders in Phase 2 validation study.

Initially 410 subjects were recruited and 3 subjects who had incomplete data were excluded from the analysis. In total, 95 oral mucosal disorders were detected amongst the 407 selected individuals: leukoplakia (34), OSF (10), lichen planus (2) and 49 with other oral mucosal abnormalities. Using all the risk factors and indicators in the risk-factor model, a ROC curve was plotted for total score assigned to each subject and the same subject's disease status as defined earlier. For OPMD excluding lichen planus (Figure 2A), a cutoff score of 12 yields maximum length to the diagonal line with an AUC of 0.87 (95% CI: 0.83–0.91), a sensitivity of 95.5%, a specificity of 75.9%, a FPR of 24.0%, a FNR of 4.5%, a PPV of 35.9% and a NPV of 99.2% (Table 5).

When all mucosal diseases are plotted against the full range of risk indicators and risk factors (Figure 2B), the same optimal cutoff score of 12 emerges, with an AUC of 0.80 (95% CI: 0.75–0.85), sensitivity of 85.3%, a specificity of 75.9%, a FPR of 24.0%, a FNR of 14.7%, a PPV of 51.9% and a NPV of 94.4% (Table 6).

## Discussion

In Sri Lanka, there is a need for developing a new strategy for the early detection of oral cancer, owing to the high morbidity and mortality associated with the current late presentation of cases (National Cancer Control Programme Sri Lanka, 2009). Moreover, recent studies have shown a dramatic increase in OPMD (Ariyawardana *et al*, 2007; Amarasinghe *et al*, 2010). In spite of the fact that oral cancer screening programmes are Government policy, there is low coverage and limited enthusiasm for oral screening amongst PHC staff (Amarasinghe, 2007). This risk-factor model has therefore been developed as a simpler and more cost-effective approach to screen high-risk people from any community with a similar risk profile as this study setting, to enable early detection of cases.

In principle, for a screening test to be considered worthwhile, the factors included in any risk-factor model need to be strongly associated with the disease(s) of concern, with some variation in exposure to these within the population (Wald *et al*, 1999). In our Phase 1 case–control study, smoking was not strongly associated with disease, but it was included in the model because of the known importance of this as a risk for upper aerodigestive tract malignancy in many populations (Chung *et al*, 2005; Ariyawardana *et al*, 2007; Morse *et al*, 2007). On the other hand, gender was not retained in the model. This is because, although male gender had an OR of 2.1 in our Phase 1 case–control study, this was not statistically significant (95% CI: 1.0–4.4), and largely reflects higher exposure to combined habits in men. In other populations, women can be the heaviest users of tobacco and betel quid, such as in Malaysian Tamils (Ministry of Health Malaysia, 2003). In the United Kingdom, for example, the male-to-female ratio for the incidence of oral cancer decreased between 1990 and 1999 (Conway *et al*, 2006), and this was attributed to a reduction in the ratio of male-to-female smokers. We cannot exclude a genetic reason for differences between the sexes, such as has been suggested in relation to oestrogen levels in a Hungarian population (Suba, 2007). On balance, we argue that it is better to quantify the risk factors themselves, rather than to confound the risk-factor model with an additional score for sex.

The results show good criterion validity, with outcomes as good as or better than many other cancer screening tests in use today (Table 7), for example, mammography and breast cancer (Ferrini *et al*, 1996; Taplin *et al*, 2008), ‘PAP smears’ and cervical cancer (van der Graaf *et al*, 1987; De Vuyst *et al*, 2005; Mayrand *et al*, 2007) and faecal occult blood and bowel cancer (Rennert *et al*, 2001; Strul and Arber, 2002; Castiglione and Zappa, 2003).

A recent study from Taiwan has generated ROC curves for individual risk factors with similar results (Yang *et al*, 2010). However, in the present work, we have combined scores for all risk factors, as this is likely to be more sensitive. The combination is additive because our data from Phase 1 revealed the synergistic effects of betel-quid chewing and alcohol use to be additive. Should subsequent studies in Sri Lanka, especially if they can contribute to a large meta-analysis, reveal multiplicative or intermediary effects, this model can be readily modified. If there are differences in another country or region, similar appropriate modifications can be made.

With a cutoff score of 12 the sensitivity, specificity and NPV of our test for the detection of OPMD is highly satisfactory. Individuals with a score at or above this should be called for a screening examination of the mouth conducted by a trained professional.

The present results show a similar sensitivity – better than 89% – to that obtained in the pioneering work performed in the 1980's in the central province of Sri Lanka, in which PHC staff conducted oral examinations for the early detection of oral cancer (Warnakulasuriya *et al*, 1984). The current FPR is, however, high, which is not a major concern, as these individuals are at high life-time risk and require habit intervention.

A strategy based on our risk-factor model should be simple to implement in terms of logistics, time and money, especially compared with existing systems, which require physical examination of whole populations. Grass-root level health volunteers or estate welfare officers can be used to select high-risk people for subsequent oral screening by trained personnel. The PHC staff can be used to supervise a programme and to arrange referral to the nearest dental clinic or organise visits by mobile clinics. The main advantage of this approach is that professional clinicians are needed only when individuals at high risk reach the clinical setting.

This approach can be integrated within the existing PHC strategies of the Sri Lankan Government or be adapted to a social marketing strategy. The latter approach has been used successfully by the anti-leprosy campaign in Sri Lanka to detect early cases of this infectious disease (Ministry of Health Sri Lanka, 2003). With such an approach, it is desirable to have a single clear message delivered consistently through all available media.

It is a common observation with screening tests that detecting those unlikely to have or to develop a disease is easier than detecting those with disease or at high risk; nevertheless, as this is the majority of a given population, the information is valuable and cost-effective (Johnson, 1991). The approach, with minor variations apposite to local lifestyles and culture, is applicable to those many countries with high incidence of oral cancer, and to the diaspora therefrom who carry their risky lifestyles with them.

### Limitations of the risk-factor model

Ethical issues arise from the FPR, because a quarter to one-third of the population will be asked to attend a mouth examination when in fact some may not have the target disease. Anxiety will be generated in subjects and their families when referral for a physical examination is made. This will also have cost implications for the patient and for providers. However, the physical discomfort involved is minimal, and a visit to a clinic provides an opportunity for habit intervention and other health screening.

Inevitably, there will also be false-negative cases, who will miss an opportunity for detection of oral disease if not referred for physical examination. For example, a minor proportion of oral cancers and of OPMD arise from factors other than betel quid, tobacco and heavy alcohol use – perhaps associated with genetic predisposition or infection with human papillomavirus or idiopathic; nevertheless, on a global scale, tobacco and other environmental risk factors vastly outweigh these as causes of the burden of disease (Axell, 1987; Herrero *et al*, 2003).

Finally, this particular model applies to the communities described herein and cannot be taken to other very different communities, for example, those in the Western world, in which areca nut habits are uncommon. The approach, however, should be widely applicable, using appropriate weightings for smoking, alcohol and other risk factors known for that particular community.

## Figures and Tables

**Table 1:** Association between OPMD and sociodemographic indicators, habits and nutritional factors in Phase 1 study

**Characteristics**	**Case (OPMD), *n* (%)**	**Control, *n* (%)**	**Crude OR (±95% CI)**	**Adjusted**^a^ **OR (±95% CI)**
*Sex*				
Female	33 (32.7)	495 (68.0)	1.0	1.0
Male	68 (67.3)	233 (32.0)	4.4 (2.8–6.8)	2.1 (1.0–4.4)
				
*Age (years)*				
30–39	21 (9.8)	194 (90.2)	1.0	1.0
40–49	28 (13.2)	184 (86.8)	1.4 (0.8–2.5)	1.1 (0.5–2.3)
50–59	33 (17.2)	159 (82.8)	1.9 (1.06–3.4)	1.0 (0.5–2.1)
⩽60	19 (9.0)	191 (91.0)	0.9 (0.5–1.7)	0.5 (0.2–1.1)
				
*Socioeconomic status*				
High	3 (3.0)	98 (97.0)	1.0	1.0
Middle	22 (9.6)	206 (90.4)	3.5 (1.0–11.9)	3.3 (0.7–15.6)
Low	76 (15.2)	424 (84.8)	5.8 (1.8–18.9)	3.0 (0.6–14.3)
BMI (continuous)	—	—	0.9 (0.8–0.9)	0.97 (0.9–1.0)
*β*-Carotene-containing total fruit and vegetable (continuous)	—	—	0.8 (0.6–0.99)	1.0 (0.8–1.3)
				
*Betel chewing*				
Never	4 (4.0)	277 (38.0)	1.0	1.0
Past and occasionally	5 (5.0)	119 (16.3)	2.9 (0.7–11.0)	2.2 (0.6–8.7)
Daily	92 (91.1)	332 (45.6)	19.2 (6.9–52.9)	10.1 (3.4–29.7)
				
*Frequency of chewing (quid per day)* b				
No chewing	4 (1.4)	277 (98.6)	1.0	1.0
⩽3	7 (5.3)	125 (94.7)	3.5 (1.1–11.3)	2.5 (0.7–8.7)
>3	85 (29.1)	207 (70.9)	27.3 (9.9–75.6)	16.2 (5.3–48.7)
				
*Alcohol drinking*				
No drinking	39 (38.6)	551 (75.7)	1.0	1.0
Past, occasional	27 (19.1)	114 (80.9)	3.3 (1.9–5.7)	1.1 (0.5–2.6)
Monthly, weekly and daily	35 (35.7)	63 (64.3)	7.8 (4.6–13.3)	2.7 (1.2–6.3)
				
*Smoking*				
Never	66 (65.3)	601 (82.6)	1.0	1.0
Ever	35 (34.7)	127 (17.4)	2.4 (1.6–3.9)	1.0 (0.5–1.7)
Total	101 (100)	728 (100)		

Abbreviations: BMI=body mass index; CI=confidence interval; OPMD=oral potentially malignant disorder; OR=odds ratio.

a OR adjusted for sex, age, socioeconomic status, *β*-carotene-containing fruits and vegetable portion, BMI, smoking, betel chewing and alcohol drinking.

b The sum does not add to the total because past and occasional chewers were excluded from the analysis.

**Table 2:** Risk factor model

**Characteristics**	**Risk score**
*Age (years)*	
15–30	0
>31	3
	
*Socioeconomic status*	
High	0
Middle and low	3
	
*Betel-quid chewing (number of quid per day)*	
Never	0
0–3	2.5
>3	16
	
*Alcohol drinking*	
Never	0
Past, occasional	1
Daily or at least weekly	3
	
*Smoking*	
Never	0
Ever	1

**Table 3:** Frequency distribution of cases and controls, applying a 12 cutoff point for the combined score and the presence of OPMD, excluding lichen planus in Phase 1 study

**Cutoff value 12**	**Cases**	**Control**	**Total**
Disease – above 12	89	235	324
No disease – <12	6	493	499
Total	95	728	823

Abbreviation: OPMD=oral potentially malignant disorder.

**Table 4:** Frequency distribution of cases and controls, applying a 12 cutoff point for the combined score and the presence of all oral mucosal diseases in Phase 1 study

**Cutoff value 12**	**Cases**	**Control**	**Total**
Disease – above 12	244	235	479
No disease – <12	57	493	550
Total	301	728	1029

**Table 5:** Frequency distribution of cases and controls, applying a 12 cutoff point for the combined score and the presence of OPMD, excluding lichen planus in Phase 2 study

**Cutoff value 12**	**Cases**	**Control**	**Total**
Disease – above 12	42	75	117
No disease – <12	2	237	239
Total	44	312	356

Abbreviation: OPMD=oral potentially malignant disorder.

**Table 6:** Frequency distribution of cases and controls, applying a 12 cutoff point for the combined score and the presence of all oral mucosal disorders in Phase 2 study

**Cutoff value 12**	**Cases**	**Control**	**Total**
Disease – above 12	81	75	156
No disease – <12	14	237	251
Total	95	312	407

**Table 7:** Sensitivity and specificity of proposed model for screening compared with test outcomes for other major cancers

**Disease and test**	**Sensitivity (%)**	**Specificity (%)**
OPMD using risk-factor model – Phase 1	93.7	67.7
OPMD using risk-factor model – Phase 2	95.5	75.9
Breast cancer screening using mammography	75–90	90–95
Cervical cancer screening using PAP test	50–85	95–99
Colorectal cancer screening with faecal occult blood test	66–85	95–97

Abbreviation: OPMD=oral potentially malignant disorder.
